# Anti-Fouling Strategies of Electrochemical Sensors for Tumor Markers

**DOI:** 10.3390/s23115202

**Published:** 2023-05-30

**Authors:** Ge Song, Hongliang Han, Zhanfang Ma

**Affiliations:** Department of Chemistry, Capital Normal University, Beijing 100048, China; sg2018@cnu.edu.cn

**Keywords:** anti-fouling, electrochemical sensor, tumor markers

## Abstract

The early detection and prognosis of cancers require sensitive and accurate detection methods; with developments in medicine, electrochemical biosensors have been developed that can meet these clinical needs. However, the composition of biological samples represented by serum is complex; when substances undergo non-specific adsorption to an electrode and cause fouling, the sensitivity and accuracy of the electrochemical sensor are affected. In order to reduce the effects of fouling on electrochemical sensors, a variety of anti-fouling materials and methods have been developed, and enormous progress has been made over the past few decades. Herein, the recent advances in anti-fouling materials and strategies for using electrochemical sensors for tumor markers are reviewed; we focus on new anti-fouling methods that separate the immunorecognition and signal readout platforms.

## 1. Introduction

Cancer has become one of the leading causes of death in many countries and regions around the world [1,2]; it usually manifests clinically in the late stages of invasion of surrounding tissues. The effects of cancer treatment are limited, and the side effects are currently significant [3]. The mortality of cancer can be effectively reduced if early diagnoses of cancer can be made, the disease development can be determined, and corresponding treatment measures can be taken in time [4]. Therefore, the early detection of cancer before symptoms appear or metastasis occurs, coupled with early interventions, can prevent and improve survival rates; this is an important research topic [5].

Tumor markers refer to a series of characteristic biochemical indicators that exist more abundantly in tumor cells or are specifically secreted by tumor cells; they can directly reflect the condition of cancer, including the metabolites of tumor cells, products of gene expression disorder in tumor cells, and products of the apoptosis and disintegration of tumor cells. They show a correlation with the occurrence and development of various types of cancers, such as prostate cancer, liver cancer, lung cancer, colorectal cancer, and so on [6,7]. Tumor markers in human blood exist in low concentrations, and their changes are related to the occurrence, development, prognosis, and cure of tumors [8,9,10]. In the early stage of cancer, the relevant markers exhibit minimal fluctuation compared to a healthy body; high sensitivity is required to detect them in real samples [11].

The development of highly sensitive, accurate, and economical tumor marker detection methods is highly important for the early diagnosis and prognostic treatment of cancer [12,13]. In the past few decades, several sensitive assays for detecting tumor markers have been developed based on the specific immune response between antibodies and tumor markers, including immunofluorescence [14], colorimetric immunoassays [15], electrochemiluminescent immunoassays [16,17,18,19], radioimmunoassays [20], photoelectrochemical immunoassays [21,22,23,24,25,26], and electrochemical immunoassays [27,28,29,30,31,32,33,34,35,36]. Among these methods, electrochemical immunosensors are widely used because of their advantages of low costs, portability, high sensitivity, and fast response speeds, which have made electrochemical biosensors an essential basic tool for clinical diagnosis in many in situ and point-of-care (POC) situations [37,38]. Electrochemical biosensors identify biological functions in processes by reading out different electronic responses, such as differences in the current, charge, potential, and impedance, which correspond to modification and stimulation processes on the electrode’s surface. Experimental results can provide important information for describing and analyzing various biological processes [39]. After decades of development, electrochemical immunosensors have attracted the attention of researchers, and significant breakthroughs have been made [40].

The composition of biological samples is very complex, as they contain a variety of biomolecules in addition to the target analyte to be measured, such as proteins, peptides, fats, and hormones. In the process of conducting immunoassays, the non-specific adsorption of coexisting species will cause false positives, false-negative, and lowered signal-to-noise ratios [41,42,43], weakening the accuracy and sensitivity of immunosensors. The fouling caused by non-specific adsorption on the electrode’s surface is one of the major problems that need to be solved in the practical applications of electrochemical immunosensors. The construction of anti-fouling electrochemical immunosensors represents a crucial step from theoretical research to practical sample detection. Thus, electrodes are commonly modified with anti-fouling material, together with antibodies.

As the immune recognition process usually occurs on the electrode’s surface, biologically complex samples have the opportunity to contact and contaminate the electrode’s surface. The anti-fouling process is mainly intended to prevent the occurrence of non-specific adsorption through the hydrophilic, electro-neutral, or steric hindrance of anti-fouling materials in the immune recognition process after the addition of biological fluid samples, such as urine, serum, or plasma. In most cases, the method used to reduce non-specific adsorption involves applying anti-fouling materials directly onto the electrode’s surface. These materials include PEG and its derivatives [44,45,46,47,48,49], zwitterionic polymers [41,50,51,52], peptides [53,54,55,56,57], and hydrogels [58,59,60,61], among others [62,63,64]. A new method has been developed in recent years, whereby the immunorecognition and signal readout platforms are separated, and the anti-fouling material is used to modify magnetic beads instead of the electrode’s surface, resulting in good conductivity of the electrode [65,66,67,68,69,70,71,72]. The immunological recognition process occurs on the magnetic beads’ surface, taking advantage of the large specific surface area of functionalized magnetic beads to increase the sites for antibody binding. Furthermore, sandwich assays are much less sensitive to fouling [42]. The non-target substances in complex samples can be completely washed away and have no chance to contact the electrode. The possibility of electrode surface contamination can be completely eliminated. Fouling resistance is the most significant feature of this method.

In this review, we introduce the most frequently used anti-fouling materials, describing their principles and giving examples. Moreover, an outlook on the future of anti-fouling electrochemical immunosensors for tumor markers is also presented.

## 2. Different Anti-Fouling Strategies Based on Anti-Fouling Materials

### 2.1. Polyethylene Glycol (PEG) and Its Derivatives

Polyethylene glycol (PEG) and its derivatives have been widely used as anti-fouling materials since the 1970s [73]. After PEG is modified on the electrode’s surface, abundant hydroxyl groups at the end of the PEG chain form a hydration layer through hydrogen bonding. The hydration layer plays an important role in surface resistance to protein adsorption [52]. The water molecules in this hydration layer are relatively firmly fixed between the electrode interface and the serum due to the action of hydrogen bonding. It is difficult for the protein to break through the hydration layer and stick to the electrode’s surface [42].

**Figure 1:**
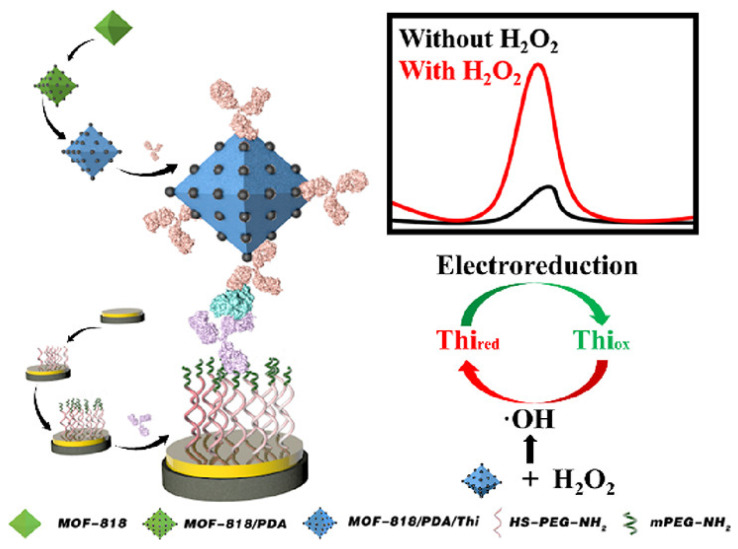
The fabrication process of the electrochemical immunosensor based on MOF-818 synergizing with an anti-fouling sensing interface. Reprinted with permission from Ref. [46], Copyright @ 2022, American Chemical Society.

Due to the poor electrical conductivity of PEG, the impedance of the modified electrode surface will increase, reducing the sensitivity of detection. The use of immunoprobes enables signal amplification and improves sensor performance through collaboration with anti-fouling sensing interfaces. Ma and his co-author designed an immunoprobe in which metal–organic framework 818 (MOF-818) served as a support to enrich the electrochemical signal. The anti-fouling sensing interface was self-assembled by HS-PEG-NH2 on gold–sulfur bonds on gold-plated GCE, and PEG-NH2 acted as a blocker to block non-reactive sites modified by antibodies. The strong hydrophilicity of PEG was utilized to form a dense hydration layer, which effectively reduced the adsorption of non-specific proteins on the electrode surface and achieved the expected anti-fouling effect. The results showed that when human serum epididymal protein 4 (HE-4), human immunoglobulin (IgG), carcinoma antigen 125 (CA12-5), and neuron-specific enolase (NSE) were selected as interferences, the corresponding currents produced were negligible [46]. The fabrication process of the electrochemical immunosensor is shown in Figure 1.

**Figure 2:**
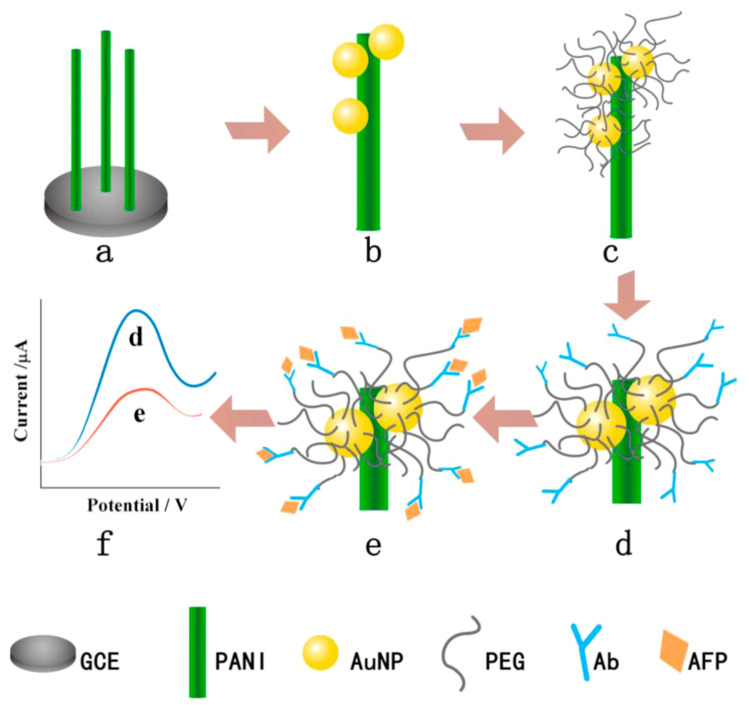
The fabrication process of the AFP immunosensor (**a**) PANI nanowires deposited on the GCE, (**b**) AuNPs electrodeposition, (**c**) PEG modification, (**d**) AFP antibody immobilization, (**e**) AFP target capturing, and (**f**) DPV current signal recording with/without AFP. Reprinted with permission from Ref. [48], Copyright @ 2016, Elsevier.

Poly(3,4-ethylenedioxythiophene) (PEDOT) and polyaniline (PANI) are widely used in electrochemical biosensors due to their stability in the oxidized state, ease of processing and excellent electrical conductivity [74,75]. They cross-linked PEG together to form the anti-fouling layer, which can effectively improve the resistance caused by modification of the anti-fouling layer. PEG can copolymerize with conductive polymers on the electrode surface [47] or be modified after the deposition of the conductive polymer [48] (Figure 2). The modification time of copolymerization is shorter, and the steps are fewer. However, attention should be paid to the content of copolymerization raw materials to generate composite materials with both anti-fouling and conductive functions. Moreover, copolymerization will cause a part of PEG to be entrapped in the conducting polymers; only the surface exposed part can exhibit anti-fouling ability with a low utilization rate. The second method requires the conductive polymer to have an anchoring functional group of PEG; otherwise, the conductive polymer surface is necessary to be modified to immobilize the bio-recognition molecules. Gold nanoparticles were deposited on PANI to assist PEG fixation, for the PANI had a few amino as anchoring functional groups [76].

**Figure 3:**
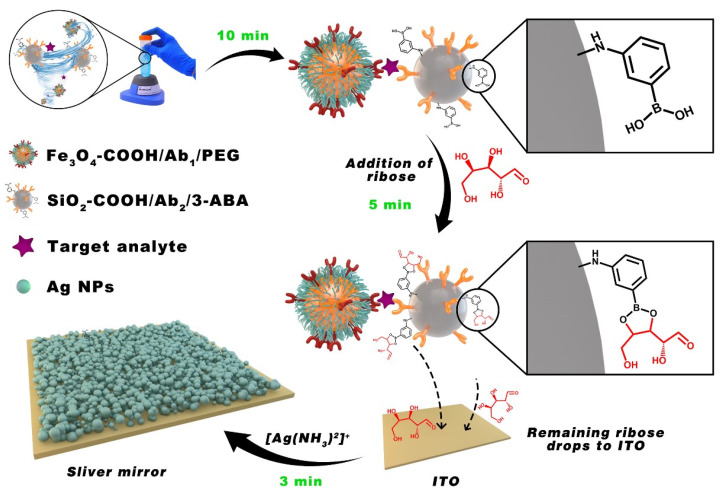
Schematic presentation of EC Sensor at zeptomolar level. Reprinted with permission from Ref. [77], Copyright @ 2022, American Chemical Society.

Due to the magnetic beads’ contact with the serum rather than the electrode surface, it is also necessary to modify the magnetic beads’ surface with anti-fouling materials to avoid the occurrence of non-specific adsorption. Zhang and co-authors reported an ultra-sensitive protein serum assay at the zeptomolar level, which combined the silver mirror reaction. The separation of immunorecognition and signal readout platforms prevented noise interference and biofouling at the electrode interface, and PEG was used to resist the adsorption of non-specific proteins (Figure 3). This biosensor was utilized to detect h-IgG and exhibited a large dynamic range from 1 μg mL^−1^ to 100 ag mL^−1^ and an ultralow limit of detection (LOD) 6.31 ag mL^−1^ (0.04 zeptomoles mL^−1^) [77].

### 2.2. Zwitterionic Materials

Complex biological samples contain charged materials, such as proteins. The protein carries a net charge due to the presence of ionized side chains on the protein surface. At suitable pH surroundings for the detection of tumor markers, charged proteins are present in the sample. The non-selectivity electrostatic adsorption possibly occurs if the electrode surface has the opposite charge to proteins. Using electro-neutral material to modify the electrode is an effective means to reduce electrostatic adsorption. Zwitterionic materials can form a hydrated layer through hydrogen bonds while being electrically neutral integrally as a whole, which is the key to its anti-fouling performance [52,78,79,80].

**Figure 4:**
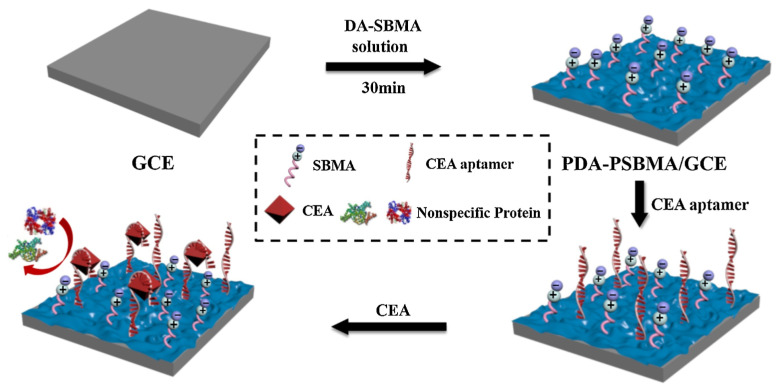
The fabrication process of the AFP immunosensor. Reprinted with permission from Ref. [50], Copyright @ 2020, Elsevier.

Poly(sulfobetaine methacrylate) (PSBMA), for instance, has possessed equivalent positive and negative charged groups, the whole molecule is electric neutrality [50], and water molecules are strongly hydrogen-bonded at its interfaces [51]. Recent research reported that SBMA and dopamine (DA) monomers could undergo polymerization on the electrode surface, forming PDA-PSBMA film to resist anti-nonspecific adsorption (Figure 4). Polydopamine (PDA) was used as a strong adhesive to stabilize PSBMA on the electrode. Aptamers or antibodies with thiol groups could attach to the PDA via the Michael addition reaction. The PDA-PSBMA interface was used to track the dynamics of ascorbic acid (AA) in the rat brain both in a normal state and in Parkinson’s disease [41]. PDA-PSBMA had shown high oxidation resistance, hydrolysis stability, and anti-fouling properties. Nevertheless, betaine polymers require complex pretreatment when used at the interface.

Yao et al. developed a regenerating electrochemical detection platform that can be regenerated in 3 min. The anti-fouling immunomagnetic beads were obtained by modifying SBMA and antibodies on magnetic beads. SBMA provided the anti-fouling function for immunomagnetic beads, and the dual interface mode reduced the non-specific adsorption as well. Squamous cell carcinoma (SCCA) was detected as the model analyte, and carbohydrate antigen 19-9 (CA19-9), alpha-fetoprotein (AFP), prostate-specific antigen (PSA), carcinoembryonic antigen (CEA), and neuron-specific enolase (NSE) were tested as interfering substances, indicating a high specificity of the biosensor. The concentration of SCCA from 1 pg mL^−1^ to 1 μg mL^−1^ possesses a linear relationship with the logarithmic value of the normalized SWV signal intensity, and the LOD of this platform was 31.20 fg mL^−1^ [81].

### 2.3. Peptides

The natural biocompatibility of functionalized anti-fouling peptides makes them become a popular anti-fouling material and widely used in an electrochemical assay for tumor markers. Due to the polar functional groups and high hydrogen bonding ability of zwitterionic charges, peptides are strongly hydrated, which is one of the reasons for their anti-fouling function. Hydrophilic and amphiphilic peptides without charge also have anti-fouling properties; for instance, the anti-fouling segment of peptides based on the EK motif was designed as electroneutrality [82,83]. The electrically neutral or hydrophilic functionalized anti-fouling peptides can be designed. All these advantages make peptides a prime candidate for biodegradable anti-fouling materials [54,55,84].

#### 2.3.1. Straight Peptides

Complex biological samples contain various enzymes. Peptides are prone to enzymatic degradation, which will reduce their anti-fouling effect. The anti-fouling performance can be improved by enhancing the resistance to enzymatic decomposition of peptides. Luo’s group designed a novel anti-fouling peptide with three D-amino acids in the N and C terminals (pD-Peptide), which made the anti-fouling peptide stable in human serum and resistant to enzymatic hydrolysis. The aptamer and pD-peptide were successively immobilized on the PANI-modified electrode with abundant amino groups. Due to the anti-enzymatic hydrolysis ability of D-amino acids at the two terminals of the peptide, the anti-fouling property of the pD-peptide-modified electrode was proved in different charge protein solutions and different concentrations of human serum samples. This modified electrode also enhanced long-term anti-fouling performance compared with normal L-peptide. This anti-fouling electrochemical biosensor demonstrated excellent selectivity with a wide linear range (from 0.1 fg mL^−1^ to 1.0 ng mL^−1^) and a low detection limit (0.031 fg mL^−1^). The result was measured with the electrochemiluminescence (ECL) assaying method used in hospitals and is closely matched, with the RSD ranging from 0.64% to 9.5% [85].

**Figure 5:**
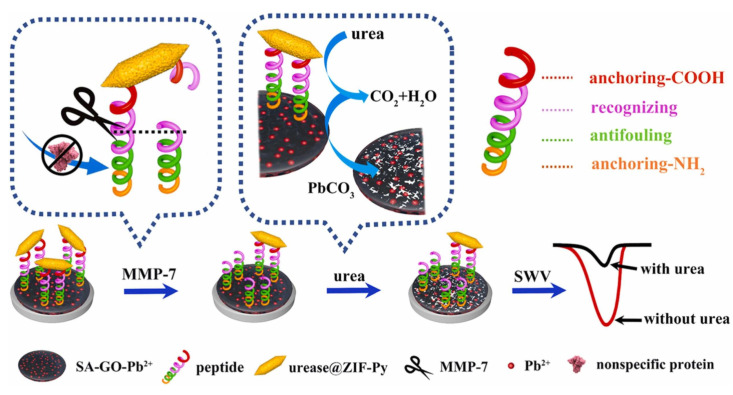
The detection process of the anti-fouling electrochemical sensor for metal matrix protease-7. Reprinted with permission from Ref. [53], Copyright @ 2022, Elsevier.

Another design idea for anti-fouling peptides is to develop the peptides with recognition sequences. The peptides which contain an electrical signal are cleaved by antigens and also have good specificity. Therefore, the electrode surface, where the aptamer was originally modified, for instance, can be used to modify the multifunctional peptide with the recognition segment. The tumor marker matrix metalloproteinase-7 (MMP-7) is a proteolytic enzyme present in serum that can specifically cleave the peptide sequence of RPLALWRSC. Based on that, an anti-fouling and sensitive biosensor was designed (Figure 5). The anti-fouling sequence was EKEKEK, which was electrically neutral to avoid non-specific adsorption. The pep/SA-GO-Pb^2+^/GCE surface was proven to have a remarkable anti-fouling property in different biological environments such as antigens (NSE, CEA, IgG, and PSA), cholesterol, amino acids, dopamine (DA), ascorbic acid (AA), and uric acid (AA). The peptides linked the hydrophilic interface with sodium alginate–graphene oxide-Pb2+ (SA-GO-Pb^2+^) gel and the novel carboxyl-rich pyrrole-doped ZIF loaded with urease (Urease @ZIF-Py). The anti-fouling effects of peptides and the cascade initiated by urease made this biosensor have a satisfactory linear relationship with the logarithm of the MMP-7 concentration range of 1 pg mL^−1^–100 ng mL^−1,^ and LOD was 24.34 fg mL^−1^ (S/N = 3) [53].

PSA is a protease tumor marker that can specifically cleave the amino acid sequence “HSSKLQ”. “KEK” amino acid sequence is electro-neutral, which acts as an anti-fouling segment to reduce non-specific adsorption, and ferrocene can link terminally to provide an electrochemical signal. Based on this idea, an electrochemical sensing interface based on the oriented self-assembly of histidine-labeled peptides induced by Ni^2+^ for protease detection was developed. This sensor had significant anti-fouling performance and can be removed by pickling to realize the reconstruction of the recognition layer, in which the reproducibility was about 80% [86].

#### 2.3.2. Peptides with Branches

**Figure 6:**
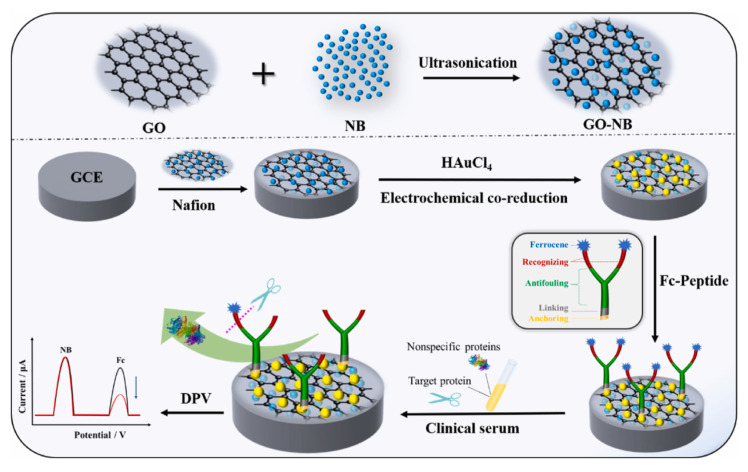
The fabrication process of the anti-fouling electrochemical biosensor based on the designed multifunctional peptide with two recognizing branches of the target IgG. Reprinted with permission from Ref. [88], Copyright @ 2022, Elsevier.

**Figure 7:**
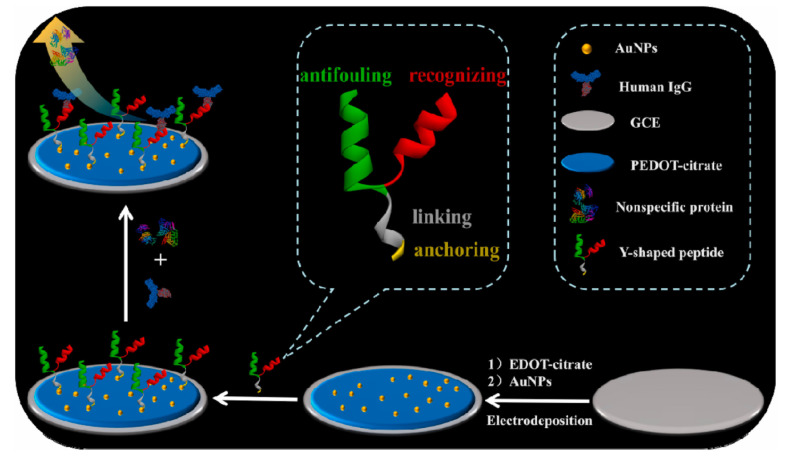
The schematic diagram for the fabrication of anti-fouling biosensors based on designed Y-shaped peptides. Reprinted with permission from Ref. [89], Copyright @ 2021, Elsevier.

In addition to the straight chain structure, anti-fouling peptides can also be designed with branches to form Y-shaped structures. The branches can be either both recognition sequences (Figure 6) [87,88] or recognition sequences with anti-fouling sequences, respectively (Figure 7) [89]. The Y-type peptide with a single recognition branch has one recognition branch and one anti-fouling branch, and its anti-fouling performance was better than that of the straight-type peptide, which was proved by the anti-fouling performances assays of BSA (negatively charged), Lys (positively charged), Mb (neutral in charge) and different concentrations of human serum. This biosensor was able to resist biofouling in various protein solutions and serum samples and could be the base for developing other biosensing systems for different targets [89]. Another research indicated that there is a synergistic effect between the two octapeptides, FYWHCLDE and FYCHTIDE, which makes a higher selectivity for IgG [90]. Based on that, an anti-fouling electrochemical sensor with dual-recognizing branch peptides was developed [87]. The detection limit of the sensor was 0.031 pg mL^−1^, which was two orders of magnitude lower than the biosensor with single-recognizing branches peptide. This biosensor could detect IgG in real biological samples, such as human serum, and the contamination of the sensing interface by complex biological media was effectively avoided. The peptide sequence of HSSKLQ can be specifically cleaved by PSA, and the GO-NB nanocomposite was modified onto the electrode surface to enhance the conductivity and specific surface area of the electrode. NB was used as an internal electrochemical probe to generate a reference signal, and the ratiometric signal of Fc/NB was used as the final sensing signal, which can provide higher sensitivity in addition to the anti-fouling function of the sensor [88].

### 2.4. Hydrogels

Hydrogels are cross-linked networks composed of hydrophilic polymers with abundant hydrogen bonds. With a large specific surface area and unique three-dimensional network structure, hydrogels can accommodate abundant modified materials to modify the electrodes. Therefore, hydrogels can load a large number of molecules with different functions and give them good conductivity, strong electrochemical signals, and excellent catalytic ability. At the same time, the micro-environment present in hydrogels can increase the stability and biological activity of biomolecules. Additionally, the high permeability of hydrogels can accelerate the transport of small molecules and ions, as well as the rapid transfer of electrons. These unique properties make hydrogels possess great potential in building electrochemical immunosensing interfaces [90,91,92,93].

**Figure 8:**
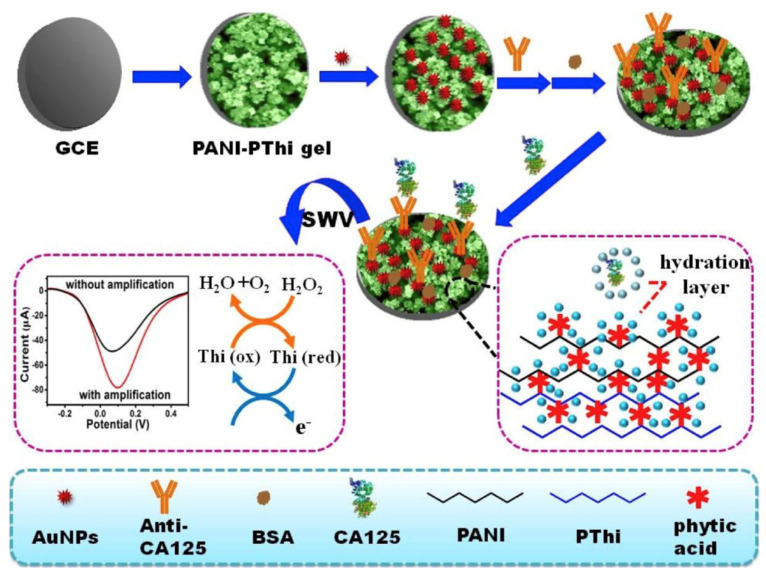
The schematic diagram of the label-free amperometric immunosensor for CA12-5. Reprinted with permission from Ref. [94], Copyright @ 2018, Elsevier.

As shown in Figure 8, a novel redox polyaniline-polythionine hydrogel (PANI-PThi gel) was fabricated as an ultrasensitive and protein-resistant label-free amperometric immunosensing platform for carcinoma antigen-125 (CA12-5). In the anti-fouling performance test, the PANI-PThi gel-modified electrode was immersed in PBS solution with different concentrations of serum for 12 h, and the current fluctuation range was not more than 4 μA, which proved that the PANI-PThi gel could significantly reduce the non-specific adsorption of proteins in human serum. Moreover, SWV curves indicated that the proposed hydrogel has a large and repeatable signal and could remain at 94.6% after a month. The current response of PANI-PThi gels can be amplified in the presence of H_2_O_2,_ which contributes to a wide linear range for the immunosensor. The result of such biosensors agreed well with ELISA in the detection of clinical serum samples [94].

**Figure 9:**
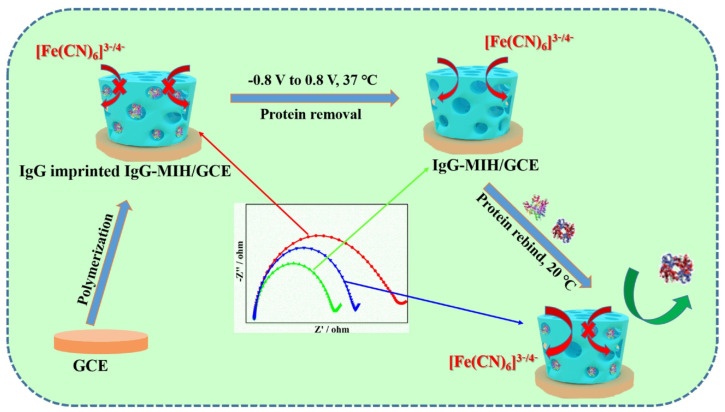
Schematic fabrication protocol of the electrochemical biosensor based on IgG-imprinted hydrogel. Reprinted with permission from Ref. [61], Copyright @ 2021, Elsevier.

An immunoplatform with high specificity and anti-fouling performance could be established in the hydrophilicity of the hydrogel coupled with the specificity of the molecular imprinting technique [95]. Luo et al. synthesized a protein-imprinted hydrogel that has temperature-responsive PNiPAAm chains. The template protein IgG can easily remove without the aid of any washing solvents at 37 °C. The structure of the IgG-MIH/GCE will revert to its initial form when the temperature is back to 20 °C. IgG-MIH/GCE surfaces were incubated in a series of complex protein-containing solutions and HeLa cell medium, indicating that the IgG-MIH/GCE surfaces had long-term stability, anti-fouling ability, and the ability to prevent cell adhesion (Figure 9) [61].

### 2.5. Bovine Serum Albumin (BSA)

Serum albumins (SAs) are one of the most widely abundant protein components in the body, both in animals and human beings. SAs are well known as a stable and widely applicable anti-fouling material to resist non-specific protein adsorption and often are used as passivation or blockers in electrochemical biosensors due to the conservation of both amino acid sequences and a three-dimensional structure [96]. Immunomagnetic beads can take advantage of the huge specific surface area when contacting complex biological samples. After the antibody was modified on the surface of the immunomagnetic beads, BSA acted as a blocking agent to block any possible remaining active sites and prevent the non-specific adsorption of proteins in complex biological samples. The resulting biosensor still has good sensitivity and specificity performance and an anti-fouling effect [66,67,68,69,70,71,97].

**Figure 10:**
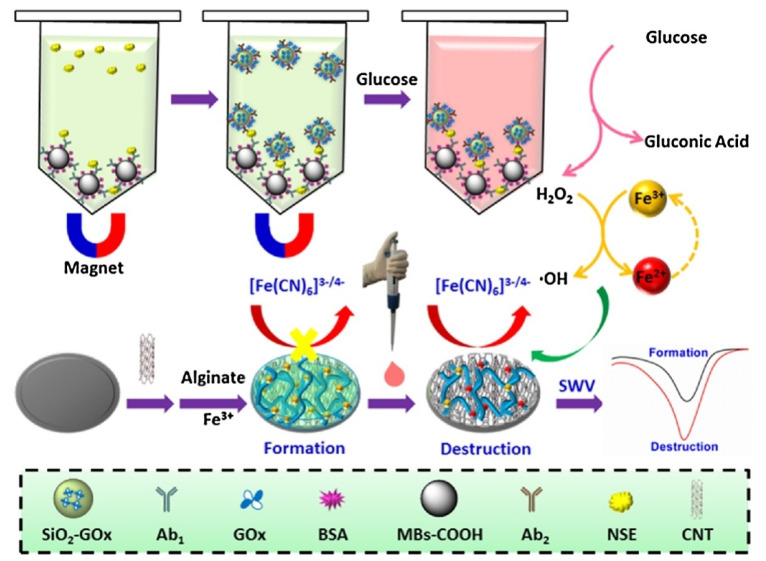
Schematic illustrations of the “smart” electrochemical sensing interface for NSE. Reprinted with permission from Ref. [68], Copyright @ 2019, Elsevier.

Yin et al. designed an enzyme-Fenton reaction that triggered the destruction of Fe^3+^ cross-linked alginate hydrogel to improve the electrochemical immunosensor, as shown in Figure 10. Silica oxide-glucose oxidase nanocomposites were used as immunoprobes to realize the high efficiency of glucose oxidation and H_2_O_2_ generation. With the continuous supply of H_2_O_2_, the Fenton reaction was triggered. Fe^3+^ transformed to Fe^2+^ and destroyed the structure of Fe^3+^-alginate hydrogel, leading to a significant decrease in the interface resistance. Under optimal conditions, neuron-specific enolase (NSE) was quantitatively detected with a wide linear range from 1 pg mL^−1^ to 100 ng mL^−1^, a low detection limit of 0.447 pg mL^−1^ [68].

### 2.6. Other Anti-Fouling Materials

**Figure 11:**
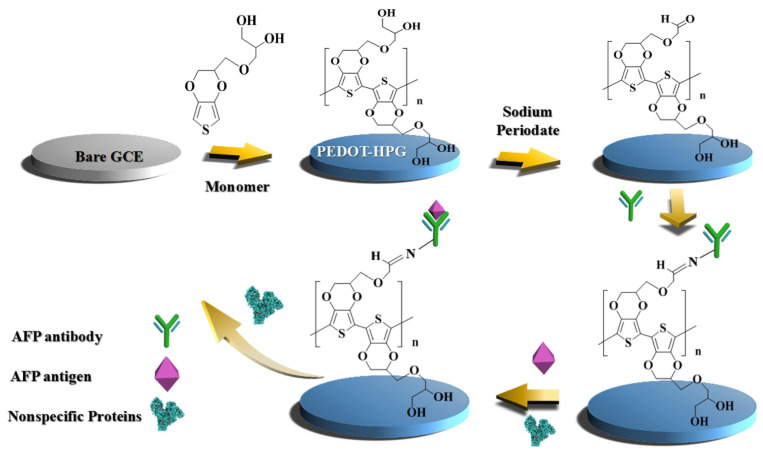
Schematic illustration of the fabrication process of the AFP biosensor with PEDOT-HPG. Reprinted with permission from Ref. [62], Copyright @ 2019, Elsevier.

Owing to its compact, globular structure with a multitude of hydroxyl groups, hyperbranched polyglycerol (HPG) has great hydrophilicity. Through the electrochemical polymerization of corresponding monomers, PEDOT was polymerized with HPG to improve the conductivity. The hydration layers were formed due to the presence of abundant hydroxyl groups on the surface of PEDOT-HPG, which inhibits the adsorption of proteins and resists non-specific cell attachment. The research showed that the biosensor had good anti-fouling ability and sensitivity with a detection limit is 0.035 pg mL^−1^ (Figure 11) [62].

**Figure 12:**
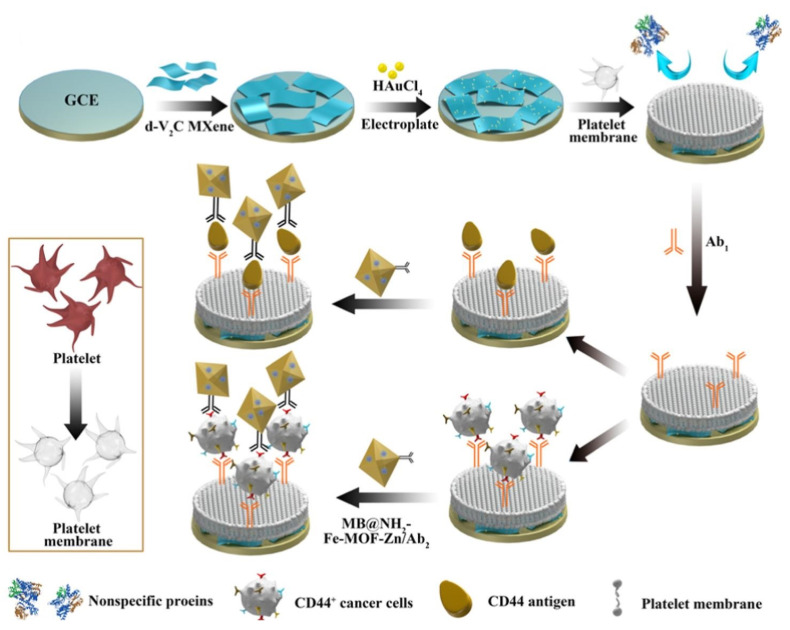
The fabrication process of the electrochemical immunosensor with anti-fouling capability for detection of CD44. Reprinted with permission from Ref. [63], Copyright @ 2022, American Chemical Society.

The cell membrane itself has good biocompatibility. Due to the structure of the phospholipid bilayer, the hydrophilic phospholipid head was exposed to the sample. In the meanwhile, the head of the phospholipid contains the negatively charged phosphate group and the positively charged quaternary ammonium group, making the whole surface electroneutrality [98]. The platelet membrane/Au nanoparticle/delaminated V2C nanosheet (PM/AuNPs/d-V2C)-modified electrode with antibacterial fouling ability depends on the steric hindrance of glycoproteins, the abundance of zwitterionic headgroups in phospholipid bilayers and the good hydrophilicity of the interface (Figure 12) [63].

Engrailed 2 (EN 2)protein is a biomarker of prostate cancer (PCa). Its isoelectric point is equal to 9.1, so EN 2 is positively charged at physiological pH (7–7.4), which inspired the authors to use positively charged histamine molecules to prevent non-specific adsorption of EN 2 on the pre-functionalized electrodes [64]. In previous reports, only three of the eleven commercially available ELISA kits passed the accuracy threshold, reflecting the difficulty of developing ELISA urine tests with adequate analytical performance [98]. As positively charged blockers, histamine molecules can effectively prevent EN 2 from non-specific adsorption on the pre-functionalized electrode due to electrostatic repulsion. The designed immunosensor is a promising alternative to ELISA assay.

## 3. Conclusions and Prospects

In conclusion, we have summarized the principle of anti-fouling materials commonly used and introduced the method which separated the immunorecognition and signal readout platforms. These anti-fouling materials are widely used for anti-fouling electrochemical sensors with the advantages of high hydrophilicity or electroneutrality. However, they have certain constraints in practical application. PEG and its derivatives were prone to oxidate into aldehydes and ethers by oxygen, resulting in polymer long-chain breakage and shedding. The deposition after the synthesis of anti-fouling conductive polymer monomers, such as SBMA-EDOT, is complex and time-consuming. Complex biological samples contain various enzymes which could make peptides enzymatic degradation. These nonconductive anti-fouling materials on the electrode surface will generate impedance to the immune layer of the electrode sensing interface (Table 1 and Table 2).

In addition to the limitations of the natural character of these anti-fouling materials, the type of non-specific adsorption also needs to be fully considered. Since electrostatic adsorption is not selective and directional, the overall electrification of other modified materials and anti-fouling materials on the electrode base should be fully considered in the design of the electrode substrate. Strictly speaking, anti-fouling peptides, for example, are electrically neutral only at the anti-fouling segment, while other amino acid sequences may be charged. Therefore, the distance between the charged part of the anti-fouling material and the complex biological samples is one of the factors affecting the anti-fouling performance.

The future trend of electrochemical biosensors is rapid detection in vivo. This requires anti-fouling sensors to have strong long-term stability and long-term anti-fouling performance, which is another big challenge in the development of anti-fouling biosensors. The ideal anti-fouling material can enhance the signal-to-noise ratio with a strong hydrophilic and electro-neutral structure, and it can also reduce the non-specific binding. After modification, it can stably exist on the electrode or immunomagnetic bead and does not affect the binding of antigen-antibody, nor does it react with antibodies or detection reagents. Most anti-fouling sensors are stable for only four weeks and need to be stored at 4 °C, and some need to be protected from oxidation by oxygen. Consequently, the development of a long-term stable anti-fouling material with hydrophilicity, electroneutrality, and strong conductivity simultaneously is particularly important. For instance, zwitterionic inorganic/organic polymers can achieve desired hydrophilic, electro-neutral structures and high resistance to enzymatic decomposition through molecular design. To improve the conductivity, zwitterionic polymers can introduce the conjugated groups or copolymerize with conductive polymers. For the method which separates the immunorecognition and signal readout platform, the prepared magnetic beads and immunoprobes individually packaged with modified or bare electrodes can be better for commercial promotion and use, which has low requirements for testing personnel.

## Figures and Tables

**Table 1:** Summary of anti-fouling materials with principles and limitations.

Anti-Fouling Materials	Strong Hydrophilicity	Electrically Neutral	Limitations
PEG and its derivatives	√	√	Prone to oxidate into aldehydes and ethers by oxygen, resulting in polymer long-chain breakage and shedding
Zwitterionic materials	√	√	Electric field-sensitive or pH-sensitive zwitterions are used under limited conditions
Peptides	√		Suffer from oxidative damage or protease degradation
Hydrogels	√		Low mechanical strength, high water content, poor freezing resistance
BSA			Cannot ensure full blocking of the active sites and may detach from the surfaces gradually

√ refers to positive in this principle.

**Table 2:** Summary of the biomarkers, anti-fouling material type, detection range, and detection limit of the anti-fouling electrochemical sensor.

Biomarker	Anti-Fouling Material	Detection Range	Detection Limit	Year	Ref.
FBP	PEG	1 × 10^−3^ to 5 × 10^2^ ng mL^−1^	2 × 10^−4^ ng mL^−1^	2019	[44]
CA72-4	PEG	1 μU mL^−1^ to 10 U mL^−1^	26.48 nU mL^−1^	2022	[46]
AFP	PEG	0.001 fg mL^−1^ to 10 fg mL^−1^	0.0003 fg mL^−1^	2016	[47]
AFP	PEG	0.01 pg mL^−1^–1.0 ng mL^−1^	0.007 pg mL^−1^	2016	[48]
CA19-9	PEG	0.00001 to 100 U mL^−1^	1.03 μU mL^−1^	2022	[49]
IgG	PEG	1 ng mL^−1^ to 100 ag mL^−1^	6.31 ag mL^−1^ (0.04 zeptomoles mL^−1^)	2022	[77]
CEA	Zwitterionic	0.01–10 pg mL^−1^	3.3 fg mL^−1^	2020	[50]
SCCA	Zwitterionic	1 pg mL^−1^ to 1 μg mL^−1^	31.20 fg mL^−1^	2022	[81]
AFP	Zwitterionic Peptide	10.0 fg mL^−1^ to 100.0 pg mL^−1^	3.1 fg mL^−1^	2017	[55]
CA 15-3	Peptides	0.01–1000 U mL^−1^	3.34 mU mL^−1^	2020	[76]
AFP	Peptides	0.1 fg mL^−1^ to 1.0 ng mL^−1^	0.03 fg mL^−1^	2021	[85]
PSA	Peptides	1 pg mL^−1^ to 100 ng mL^−1^	11.8 fg mL^−1^	2023	[86]
IgG	Peptides	0.1 pg mL^−1^ to 0.1 mg mL^−1^	0.031 pg mL^−1^	2022	[87]
PSA	Peptides	5.0 pg mL^−1^ to 100 ng mL^−1^	1.26 pg mL^−1^	2022	[88]
IgG	Peptides	100 pg mL^−1^ to 10 μg mL^−1^	32 pg mL^−1^	2021	[89]
MMP-7	Peptides	0.1 pg mL^−1^–100 ng mL^−1^	24.34 fg mL^−1^	2022	[53]
BRCA 1	Peptides	1.0 fM to 10.0 pM	0.3 fM	2017	[56]
MCF-7 cancer cells	Peptides	50–10^6^ cells mL^−1^	17 cells mL^−1^	2022	[57]
HSA	Hydrogels	10^−6^–5 × 10^−2^ ng mL^−1^	0.03 × 10^−6^ ng mL^−1^	2021	[58]
PSA	Hydrogels	1.0 fg mL^−1^ to 100 ng mL^−1^	0.09 fg mL^−1^	2019	[59]
NSE	Hydrogels	0.01 to 1000 ng mL^−1^	4.6 pg mL^−1^	2018	[60]
IgG	Hydrogels	0.5–200.0 ng mL^−1^	0.03 ng mL^−1^	2021	[61]
NSE	Hydrogels	1 pg mL^−1^ to 200 ng mL^−1^	0.26 pg mL^−1^	2017	[92]
CYFRA21-1	Hydrogels	50 fg mL^−1^ to 100 ng mL^−1^	38 fg mL^−1^	2017	[93]
CA125	Hydrogels	0.0001 U mL^−1^ to 1 kU mL^−1^	0.00125 U mL^−1^	2018	[94]
SCCA	BSA	1 mg mL^−1^ to 1 pg mL^−1^	1.504 fg mL^−1^	2022	[65]
CYFRA21-1	BSA	10 fg mL^−1^ to 1 μg mL^−1^	3.175 fg mL^−1^	2020	[66]
PSA	BSA	1.0 × 10^−3^–1.0 × 10^2^ ng mL^−1^	37.27 fg mL^−1^	2021	[67]
NSE	BSA	1 pg mL^−1^ to 100 ng mL^−1^	0.447 pg mL^−1^	2019	[68]
HE4	BSA	1 pg mL^−1^ to 100 ng mL^−1^	0.302 pg mL^−1^	2021	[69]
HIF-1α	BSA	0.25–10.0 ng mL^−1^	76 pg mL^−1^	2020	[70]
HER 2-ECD	BSA	5.0 and 50 ng mL^−1^ 50 and 100 ng mL^−1^	2.8 ng mL^−1^ 3 cells mL^−1^	2020	[71]
CA 125	BSA	0.1 mU mL^−1^ to 500 U mL^−1^	0.048 mU mL^−1^	2019	[97]
AFP	Polyglycerol	0.10 pg mL^−1^–1.0 ng mL^−1^	0.035 pg mL^−1^	2019	[62]
CD44 CD44 Cancer Cell	Cell Membrane	0.5 ng mL^−1^ to 500 ng mL^−1^ 10^3^ to 10^6^ cells mL^−1^	1.4 pg mL^−1^ 140 cells mL^−1^	2022	[63]
EN 2	Histamine	10^−5^ ng mL^−1^ to 1 μg mL^−1^	10^−5^ ng mL^−1^	2022	[64]

## Data Availability

Not applicable.
